# Gender-Dependent Changes in Time Production Following Quadrato Motor Training in Dyslexic and Normal Readers

**DOI:** 10.3389/fncom.2018.00071

**Published:** 2018-08-29

**Authors:** Tal Dotan Ben-Soussan, Joseph Glicksohn

**Affiliations:** ^1^Research Institute for Neuroscience, Education and Didactics, Patrizio Paoletti Foundation for Development and Communication, Assisi, Italy; ^2^Department of Criminology, Bar-Ilan University, Ramat Gan, Israel; ^3^The Leslie and Susan Gonda (Goldschmied) Multidisciplinary Brain Research Center, Bar-Ilan University, Ramat Gan, Israel

**Keywords:** time production, quadrato motor training, dyslexia, gender difference, time and motion studies

## Abstract

Time estimation is an important component of the ability to organize and plan sequences of actions as well as cognitive functions, both of which are known to be altered in dyslexia. While attention deficits are accompanied by short Time Productions (TPs), expert meditators have been reported to produce longer durations, and this seems to be related to their increased attentional resources. In the current study, we examined the effects of a month of Quadrato Motor Training (QMT), which is a structured sensorimotor training program that involves sequencing of motor responses based on verbal commands, on TP using a pre-post design. QMT has previously been found to enhance attention and EEG oscillatory activity, especially within the alpha range. For the current study, 29 adult Hebrew readers were recruited, of whom 10 dyslexic participants performed the QMT. The normal readers were randomly assigned to QMT (*n* = 9) or *Verbal Training* (VT, identical cognitive training with no overt motor component, and only verbal response, *n* = 10). Our results demonstrate that in contrast to the controls, longer TP in females was found following 1 month of intensive QMT in the dyslexic group, while the opposite trend occurred in control females. We suggest that this longer TP in the female dyslexics is related to their enhanced attention resulting from QMT. The current findings suggest that the combination of motor and mindful training, embedded in QMT, has a differential effect depending on gender and whether one is dyslexic or not. These results have implications for educational and contemplative neuroscience, emphasizing the connection between specifically-structured motor training, time estimation and attention.

## Introduction

Timing deficits in dyslexia include those concerned with time estimation (Nicolson et al., 1995; Ramus et al., 2003; Hölzel et al., 2011), rhythm tapping (Wolff et al., 1990; Wolff, 2002), detecting complex timing patterns (Kujala et al., 2007), rapid temporal processing (Tallal et al., 1993), auditory temporal sensitivity (Witton et al., 1998) and visual motion detection (Talcott et al., 2000). Consequently, dyslexia-related timing deficits have at different times been hypothesized as underlying dyslectics’ visual and auditory perception problems, motor coordination problems and fluency and automatization problems (Nicolson et al., 2001), all of which have been proposed as adversely affecting the development of language and literacy skills (Overy et al., 2003). The full extent of the timing deficits is yet to be established, but suggests the need for further investigation.

A critical factor here seems to be the cerebellum, which is implicated in both timing functions (Ivry and Hazeltine, 1992; Ivry, 2000, p. 187; Ivry et al., 2002; Rubia, 2006) and in dyslexia (Reynolds et al., 2003; Kujala et al., 2007; Reynolds and Nicolson, 2007; Ben-Soussan et al., 2014a). Thus, cerebellar deficits are thought to affect articulation and working memory, due to deficits in timing which interfere with automatization of learning (Thomas and Karmiloff-Smith, 2002; Overy et al., 2003; Ram-Tsur et al., 2013).

In fact, cerebellar oscillatory function and its role in motor acquisition and timing have long been acknowledged (Andres et al., 1999; Swinnen, 2002; De Zeeuw et al., 2011). Given that impaired motor skills are often observed in dyslexics, some researchers have attributed dyslexics’ cognitive and motor deficiencies to abnormal development and functioning of the cerebellum (Nicolson et al., 1995, 2001). These findings have led to the claim that the role of the cerebellum is not limited to regulating the timing, rate, force, rhythm and accuracy of movements, but also to the speed, capacity, consistency and appropriateness of cognitive processes (Schmahmann, 2004; Hölzel et al., 2011; Buckner, 2013).

Ben-Soussan et al. (2015) have recently presented a general model tying cerebellar function to cognitive improvement, by means of a particular form of motor training, which might be viewed as meditation-in-action, namely Quadrato Motor Training (QMT). QMT has been found to increase creativity, reflectivity and spatial cognition (Ben-Soussan et al., 2013, 2014b, 2015), as well as to increase neuronal synchronization and connectivity, especially within the alpha (8–12 Hz) range (Lasaponara et al., 2017). In addition, a month of daily QMT was found to improve reading and increase cerebellar alpha oscillations in dyslexic adults (Ben-Soussan et al., 2014a). Recently, QMT was further found to increase fractional anisotropy (FA) in tracts related to sensorimotor and cognitive functions and mindfulness, including the corticospinal tracts, anterior thalamic radiations and uncinate fasciculi, as well as in the left inferior fronto-occipital, superior and inferior longitudinal fasciculi (Piervincenzi et al., 2017), reflecting better white matter integrity as a result of greater intravoxel coherence of fiber orientation, axon density and diameter and/or myelination (Beaulieu et al., 1996; Sen and Basser, 2005; Caminiti et al., 2013).

Let us contrast a hypothesized QMT-based improvement in the functioning of dyslexic adults to that found for another movement-based form of training, which has also reported an improvement in reading fluency for dyslectic participants. Reynolds et al. (2003) study was conducted on dyslectic pupils, reporting improvements in balance, dexterity and reading. As they subsequently reported in a follow-up study (Reynolds and Nicolson, 2007), both dyslexic and non-dyslexic children benefited from the training, while alternative hypotheses raised by critics of the original study (e.g., based on potential artifacts, such as a Hawthorne effect) could be ruled out. In these studies, training was comprised of a home-based exercise program. In the study we report below, our QMT is also a home-based motor-exercise program. We do, however, also employ a home-based verbal-exercise program, as a suitable control.

Our present question concerns the hypothesized effect of QMT on time perception (specifically, time production, TP) in dyslectic adults. In a recent study (Ben-Soussan et al., 2017), normal reading participants reported a number of changes in their time perception during QMT, including: “an elongation of time, after a while, I had time to move from point to point, I didn’t have to be in a hurry. I was faster and the exercise was slower”; “In the last day, when I finish, the precision—I can do many more things in a time, which I didn’t think I can do.” While QMT has thus been found to affect the subjective experience of time, the effects of QMT on TP have yet to be examined. Given that QMT is viewed as meditation-in-action, one can refer to the literature on meditation and time perception to develop a working hypothesis.

Meditation has been found to lead to a relative overestimation of target durations in passing (Glicksohn, 2001; Berkovich-Ohana et al., 2012; Kramer et al., 2013). Longer produced durations may be explained by a decrease in arousal (due to the decrease in the pacemaker speed of the internal clock), and an increase in size of the subjective time units (Glicksohn, 2001). QMT, viewed as meditation-in-action, should, like other forms of meditation, therefore lead to longer produced durations. We further consider gender, given that males usually make relatively longer TPs (Block et al., 2000, p. 1341; Zakay and Block, 1997, p. 13; Glicksohn and Hadad, 2012), and that male and female dyslectics may differ in the neurocognitive underpinnings of their dyslexia (Lambe, 1999). Hence, we expect to see a lengthening of TP (post—pre QMT), especially so for males. We further expect to see such effects for dyslectic individuals. If there is a gender-dependent change in TP in dyslectics, this would lend further support for searching for gender-dependent patterns of neural activity during this specific task of TP (which usually involves chronometric counting; Glicksohn and Hadad, 2012), as well as among other tasks involving auditory processing (Lambe, 1999, p. 532).

Given that our two reviewers expressed concern that because our participants were probably engaged in chronometric counting (as we, ourselves, have suggested), we might have compromised our study of TP, we shall take this opportunity to engage in debate about this issue. Some researchers argue that chronometric counting should be discouraged (e.g., Mimura et al., 2000; Kladopoulos et al., 2004); others argue that this should be encouraged (e.g., Miró et al., 2003; Myers and Tilley, 2003). Some researchers specifically request their participants to engage in counting so that the same strategy is employed by all participants (e.g., Perbal et al., 2003; Coelho et al., 2004). Counting is a natural strategy to employ in a task of TP; and, as Fetterman and Killeen (1990, p. 766) argue, “The ubiquity of the practice calls into question experimental psychologists’ attempts to prevent or interfere with subjects’ counting strategies as a means of eliciting ‘uncontaminated’ temporal judgments…” Counting is undeniably a timing task (Brown et al., 2013); both timing without counting, and timing with counting, seem to be correlated to a fair degree (Bartholomew et al., 2015). Furthermore, techniques used to prevent counting may “‥.be distracting and introduce extraneous variables that can obscure effects specifically related to timing mechanisms” (Gaudreault and Fortin, 2013, p. 598). Consider, for example, the recent study reported by Schreuder et al. (2014). They employed three target durations of 1.33, 1.58 and 2.17 min, each to be produced in their TP task. As they write (p. 3), “we wanted to use intervals that exceeded 1 min, as these seem harder to produce because participants need to concentrate for a longer period of time.” To prevent counting they required their participants to remember, in parallel, an 8-character password (e.g., Z2Hx89bS). There are two problems, to our mind, with this procedure. First, these target durations are beyond an outer bound of 100 s for what would be considered to be time perception; as Wackermann (2007, p. 20) suggests, beyond this upper bound “time is merely cognitively (re)constructed, not actually experienced or “perceived,” a fact that is frequently ignored by contemporary time perception research.” Second, one does not usually try to retain in memory an 8-character password. Hence, what exactly is being investigated in this particular task of TP? In the present study, our participants were most probably employing chronometric counting as a natural strategy, hence were involved in timing *per se*, and not in adopting what might well be for them a suboptimal and unfamilar strategy (not counting). Given our interest in the performance of our dyslectic participants, in particular, this seems to be ecologically wise.

## Materials and Methods

### Participants and Design

For the current study, 29 adult Hebrew readers (19 women (F) and 10 men (M), mean age ± SD: 28 ± 5) were recruited, of whom 10 were dyslexic. The normal readers were randomly assigned to QMT (*n* = 9; 7 F + 2 M) or *Verbal Training* (VT, *n* = 10; 7 F + 3 M); the dyslexics were assigned to QMT (5 F + 5 M). The study was approved by the ethics committee of Bar-Ilan University. Upon entering the lab, all participants gave written informed consent. The study included three phases: pre-training assessment (Day 1), 28 days of daily training, and post-training assessment of TP. Pre and Post-training assessment took place at the lab. On the other training days, the participants performed the task at home. Compliance was controlled using a diary and daily recording of the training using a webcam. In addition, a semi-structured oral interview regarding QMT-induced experience was conducted, which included three open-ended questions regarding the participant’s physical, emotional and cognitive experiences during and following QMT (Ben-Soussan et al., 2017).

### The Training Groups

#### Quadrato Motor Training (QMT)

The QMT group practiced the QMT in full. The QMT requires standing at one corner of a 0.5 m × 0.5 m square and making movements to different corners of the square in response to verbal instructions given by an audio tape recording indicating the next corner to which the participant should move. There are three optional directions of movement, and the movement is always in one step. We used a specific sequence of movements provided by Patrizio Paoletti, founder of the QMT program, translated from Italian to Hebrew by the first author. Each movement can be forward, backward, left, right, or diagonal. The instructions direct participants to keep the eyes focused straight ahead, hands loose at the side of the body. They are also told to immediately continue with the next instruction and not to stop due to mistakes. At each corner, there are three possible directions to move (for example, from corner 1 the participant can move to corner 2, to corner 3 or to corner 4). The training thus consists of 12 possible movements (3 directions × 4 corners): 2 forward, 2 backward, 2 left, 2 right and 4 diagonals. The participant is required to move from one corner to another according to the number on the recording. For example, if the sequence required is 1, 2, 1, 2, 1, 2, 3, 2, 4, 3, 1…. this means moving to the first corner, then to the second, then back to the first, and so on.

**Figure 1:**
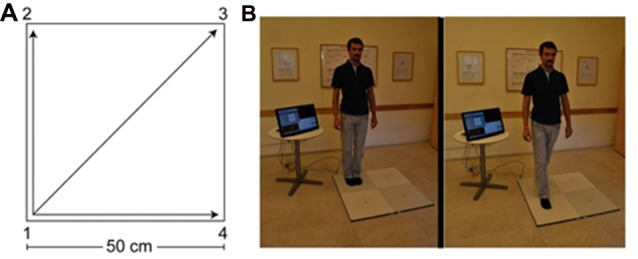
The Quadrato Motor Training (QMT).** (A)** A graphical illustration of the QMT. **(B)** A participant during the QMT while waiting for the next instruction (left) and following the instruction (right). Written consent was obtained from the individual for the publication of this image.

The practice comprised 69 commands (23 sequences of movements that last ~88 s; with a ~25 s interval between each set of 23 commands for calibration). Thus, in total the whole QMT session lasted ~6 min. Each movement has two instructions: the starting current position and the target position (“one four” means move from corner 1 to corner 4). Between noting the starting current position and the target position there was a randomized Inter-Stimulus Interval (ISI) of between 1,100 ms and 1,300 ms. ISI between trials (namely, between the previous target position and the next trial) was a randomized ISI of between 2,300 ms and 2,650 ms; see Figure 1.

In the current study we aimed at controlling limb velocity, by using a movement sequence comprising a total of 69 instruction steps, paced at a rate of 0.5 Hz (similar to a slow walking rate), which was the same for all participants. We also controlled for the decision regarding the responding limb by instructing participants to begin all movements with the leg closest to the center of the square.

#### Verbal Training (VT)

The VT group stood 1 m in front of the square, but did not move on the corners of it. Instead, their instructions were to respond to the taped commands verbally by stating what direction of movement would be required in order to reach the corner specified by the command. For a movement from corner 1 to corner 2, they were required to say “straight;” for a movement from corner 1 to corner 3, they were required to say “diagonal.” The following is a list of all possible combinations and the appropriate response: 1–2, 4-3, “straight;” 2-1, 3–4, “back;” 1–3, 4-2, 3-1, 2–4, “diagonal;” 1–4, 2–3, “right;” 4-1, 3-2, “left.”

### Time Production (TP) Task

Four target durations of 4, 8, 16 and 32 s served for the TP task. The participant was required to remain with eyes closed while producing each of these target durations by pressing a finger button (Glicksohn, 1996) for the required period of time. Each target interval was produced twice, the target durations being presented in random order to the participant. Produced (*P*) and target (*T*) durations (in seconds) were log-transformed (to base 2), with required durations rendering thereby a linear scale ranging between 2 and 5, with a midpoint value of 3.5; produced duration was then regressed on required duration. We look at three dependent measures: (1) mean log(*P*); (2) the *slope* of log(*P*) regressed on log(*T*); and (3) the *intercept* of that regression line (Glicksohn and Hadad, 2012).

## Results

**Figure 2:**
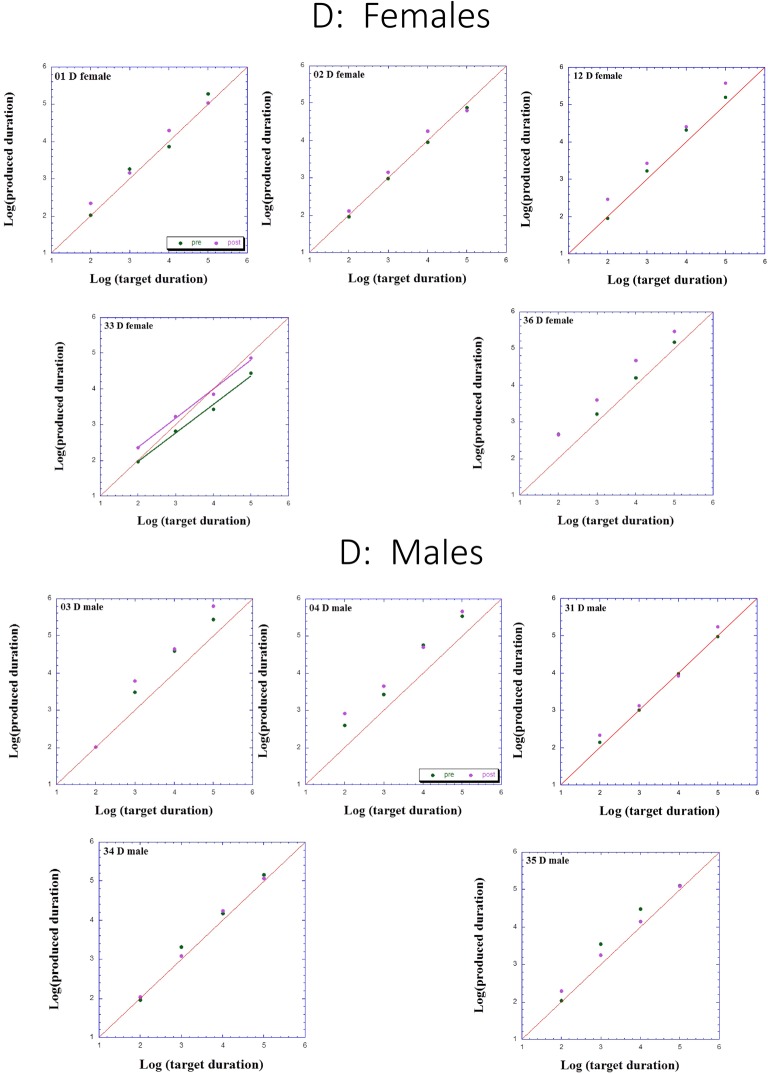
Individual log-log plots of produced duration of the dyslectics assigned to QMT group (D), as a function of target duration, blocked according to Gender.

**Figure 3:**
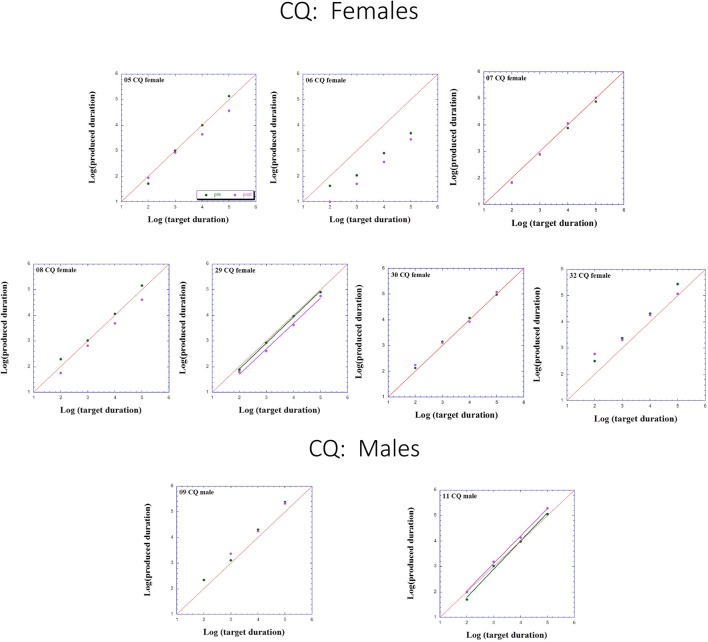
Individual log-log plots of produced duration of the controls assigned to QMT (CQ) as a function of target duration, blocked according to Gender.

**Figure 4:**
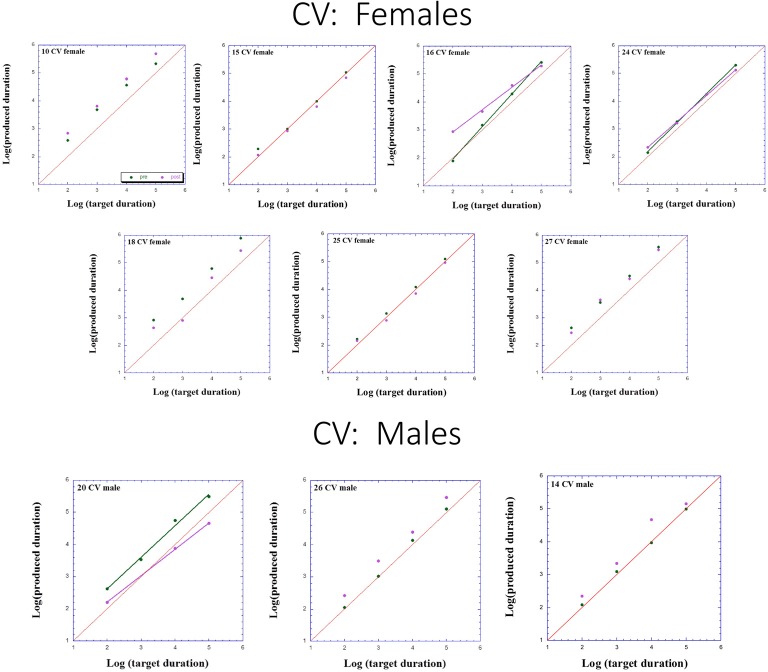
Individual log-log plots of produced duration of the controls assigned to Verbal Training (VT) control (CV) as a function of target duration, blocked according to Gender.

Figures 2–4 presents individual log-log plots of produced duration as a function of target duration, blocked according to Group and Gender. For the controls assigned to VT (CV), one may note the essential linearity of the data in the log-log plot. We have fitted the linear regression lines for one CV male, and for two CV females, to exemplify this. The diagonal in each plot indicates what would be veridical TP (i.e., produced time = target duration). Note that for two of the CV male participants, produced duration post-training is longer than that of pre-training, and for the third male, the opposite is the case. Given this small group size, these opposite trends will easily cancel out, leaving no clear post-pre difference in TP, as we will subsequently show. For the CV females, one notes an increase in produced duration post-training for two participants, a decrease in produced duration post-training for three participants, and no noticeable change post vs. pre for the remaining two participants in this group. Again, this will result in a canceling out of effects, as with the male participants.

Turning to the participants assigned to QMT, we note that for the controls (CQ), one of the two males produces longer durations post-training (as can be seen on comparing the regression lines), while four of the females produce shorter durations post-training, and for the remaining three there is no noticeable change post vs. pre. This is a clear effect for Gender, for these controls, as we will subsequently show. In contrast, when looking at the dyslectic participants, one notes that three of the five males produce somewhat longer durations post-training, and three of the five females produce markedly longer durations post-training. We turn now to a formal analysis of these trends, using analysis of variance (ANOVA).

**Figure 5:**
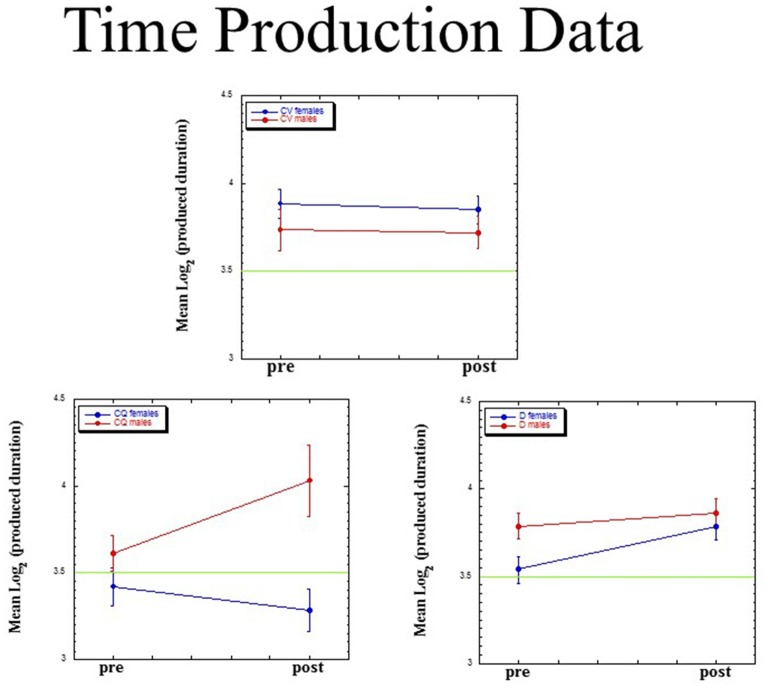
Mean (±SE) values for mean log(*P*) as a function of Group (dyslectics assigned to QMT, D; controls assigned to QMT, CQ and controls assigned to VT, CV), Gender (male, female) and Time (pre, post).

For each of our three dependent measures (mean, slope, intercept), we ran a Group (dyslectics assigned to QMT, controls assigned to QMT, controls assigned to VT) × Gender (male, female) × Time (pre, post) ANOVA, adopting the Greenhouse-Geisser *p*-value for each effect. Figure 5 presents mean (±SE) values for mean log(*P*). The three-way interaction for this measure was significant (*F*_(2,23)_ = 3.85, *MSE* = 0.068, *p* < 0.05). There was no main effect for Gender (*F*_(1,23)_ = 1.71, ns), Group (*F*_(2,23)_ < 1) or Time (*F*_(1,23)_ = 2.95, *p* = 0.10), and no two-way interactions.

We found a significant lengthening of produced time for the female dyslectics following QMT (*t*_(4)_  =  −3.80, *p* < 0.05; *n* = 5), in contrast there was a decrease in produced time for the control females following QMT (*t*_(6)_  =  2.56, *p* < 0.05; *n* = 7). No such difference was found in the VT group, either for females (*t*_(6)_  =  0.28, ns; *n* = 7), or for males (*t*_(2)_  =  0.05, ns; *n* = 3).

We ran a comparable ANOVA, this time with log(*P*) comprising a profile of four mean values for each of the four target durations (Target Duration). In this analysis, we uncovered a Target Duration × Gender interaction (*F*_(3,63)_ = 3.86, *MSE* = 0.036, *p* < 0.05), as well as the expected main effect for Target Duration (*F*_(3,63)_ = 1,481.26, *MSE* = 0.036, *p* < 0.001). In short, increasing target duration results in increasing produced duration, for both male and female participants, as one would expect; furthermore, for the longer target durations, male participants produce longer durations than do female participants, while for the specific target duration of 2 s, females produce longer target durations than males. Given that these effects are not dependent on Group, our focus on mean log(*P*) and on the three-way Group × Gender × Time interaction for this is supported.

Turning to the other two measures, we found no three-way interaction for the slope (*F*_(2,23)_ < 1) nor for the intercept (*F*_(2,23)_ < 1). Mean slope values range between 0.92 and 1.06, hence do not deviate from an expected slope of 1.00 (Glicksohn and Hadad, 2012), while mean intercept values range between −0.11 and 0.59. For the slope, there was no main effect for Gender (*F*_(1,23)_ = 3.17, *p* = 0.09) or Group (*F*_(2,23)_ < 1), and no two-way interaction. For the intercept, there was also no main effect for Gender (*F*_(1,23)_ < 1), but there was a main effect for Group (*F*_(2,23)_ = 3.58, *MSE* = 0.185, *p* < 0.05), whereby the controls assigned to VT had a higher mean intercept (0.461) than the dyslectics assigned to QMT (0.321) and the controls assigned to QMT (0.016).

There were no baseline differences in TP between dyslexic and normal readers for any of the three measures. Specifically, for mean log(*P*), slope and intercept, there was no main effect for Group (*F*_(2,23)_ = 1.56, 0.11, 1.73, respectively, all ns), for Gender (*F*_(1,23)_ = 0.55, 2.18, 0.53, respectively, all ns) nor their interaction (*F*_(2,23)_ < 1 for each).

### First-Person Reports

The semi-structured interview revealed that eight participants from the QMT group reported having increased attention and relaxation. More specifically, participant 03, a dyslexic male, reported: “I had to focus that the inner leg will be first but with practice it became less hard. In the beginning, I really had to concentrate for that. Maybe more balance and equilibrium.” Participant 34, a dyslexic male, reported having “more sharpness. Things are retrieved faster. More focused. Yes, it contributes to focus, you have focus.” Participant 33, a dyslexic female, reported having more “Attention, and listening more what people say. Not just hearing the voice but the listening.” In addition, two dyslexic and two normal readers reported a sense of relaxation and calmness following the training. Participant 1, a dyslexic female, reported: “I am calmer. I don’t know if it is because of it, but in some place my stress decreased from all things and their meaning, let’s say if I don’t find an apartment, I will go abroad, it’s not critical. Acceptance.” Participant 31, a dyslexic male, reported “relaxation as a result of the training. As a result of the relaxation, looking at the decisions in a more reasonable or concentrated way. The practice felt a bit meditative”. Participants 08 and 06, normal female readers, reported “feeling calmer;” and “feelings of relaxation and calmness. It has a bit of an effect like meditation. It enters into a state of mind that I should do the experiment. And also when my thoughts wandered, you need to be focused and to the thing to keep me in the frame,” respectively.

The only participant who reported being more aroused following the training was actually from the verbal control group: “I felt two things. One, is that when I am tired, I am more concentrated during the training and there were times I really awoke after.”

## Discussion

Time estimation is an important component of the ability to organize and plan sequences of actions as well as cognitive functions. While attention deficits are accompanied by short TPs, expert meditators have been reported to produce longer durations, related to their increased attentional resources. In the current study, we examined the effects of a month of QMT, a structured sensorimotor training program that involves sequencing of motor responses based on verbal commands, on TP using a pre-post design. Our results demonstrate that in contrast to the controls, longer TP was found following 1 month of intensive QMT in the female dyslexic group, while shorter TP was found for the control females following QMT. We suggest that this may be related to three mediating inter-related mechanisms, including enhanced attention resulting from QMT, better working memory and better cerebellar functioning. The semi-structured interview confirmed this hypothesis and revealed that participants from the QMT group reported having increased attention and relaxation. This is in line with our previous report (Ben-Soussan et al., 2017).

The involvement of the cerebellum in cognition has been overshadowed by years of focus on its motor role. Yet, the cerebellum, possibly but not exclusively through its connections with frontal and prefrontal areas, contributes to cognition, learning and language (Beaton and Mariën, 2010; Pesce and Ben-Soussan, 2016), leading also to the notion of the linguistic cerebellum (Jansen et al., 2005; Stoodley and Schmahmann, 2009). More specifically, it has been suggested that cerebellar dysfunction may be involved in dyslexia due to the cerebellum’s role as an oscillator, producing synchronized activity within neuronal networks, including sensorimotor networks critical for reading, timing and attention (Buhusi and Meck, 2005; Ben-Soussan et al., 2014a).

Within an internal-clock framework, a change in attentional resources can result in longer perceived duration (Kramer et al., 2013). Such a practice-enhanced attention results in better working memory (Davis and Hayes, 2011), which is a main deficit in dyslexics (Jeffries and Everatt, 2004; Smith-Spark and Fisk, 2007). In turn, working memory is closely related to the cerebellum (Justus and Ivry, 2001; Ravizza et al., 2006). In fact, it has been suggested that the cerebellar impairments in dyslexia, which are linked to reduced articulation speed, may lead to impaired working memory, and in turn to the language impairments (Nicolson et al., 2001).

In addition to the centrality of phonological mechanisms in dyslexia, recent evidence also supports an important role for attentional mechanisms (Shaywitz and Shaywitz, 2008; Shaywitz et al., 2017). The lengthening of TP following QMT in the female dyslexic group could be related to increased attention and activation of the cerebellum. In fact, QMT was previously found to increase mindfulness and attentional effort (Ben-Soussan et al., 2017), as well as to improve white matter integrity of neuronal pathways related to attention and learning (Piervincenzi et al., 2017) in normal readers. Furthermore, given that participants predominantly employ chronometric counting when engaging with our TP task (Glicksohn and Hadad, 2012), the (right) cerebellum was surely activated (Tracy et al., 2000; O’Leary et al., 2003; Hinton et al., 2004). QMT, similar to other mindfulness training, involves deliberately staying in the present moment (Kramer et al., 2013; Ben-Soussan et al., 2014b). We note that mindfulness meditation trains attentional skills and produces increased attentional resources (Lutz et al., 2008).

The shortening of TP for the control females following QMT could be related to their induced arousal as a consequence of the motor training, speeding up the internal clock rate, hence leading to a shortening of TP (Ozel et al., 2004). In fact, a similar trend has been previously observed on using MEG, wherein both dyslectic and control groups improved reading performance; cerebellar alpha oscillations increased in the dyslexic group, while the opposite trend occurred in the normal reader group (Ben-Soussan et al., 2014a).

A common element of many theories related to the cause of dyslexia is the conviction that timing skills, and particularly rapid timing skills and motor timing skills, are a fundamental problem area. In fact, it has been previously suggested that a deficit in rapid temporal processing can cause specific auditory perception problems, leading to specific phonological perception problems (Tallal et al., 1993). Yet, in the current study we did not find baseline differences in TP between dyslexic and normal readers in any of the three measures.

We expected to see a lengthening of TP (post—pre QMT), especially so for males. In contrast to our hypothesis, we observed a lengthening of TP, for the female participants. A trend of a lengthening of TP occurred for both QMT groups, in contrast to the VT control group, yet this lengthening of TP was not statistically significant for the males, probably due to the small number of these participants. We have detected an interaction involving gender, whereby the hypothesized lengthening of TP for both QMT groups is found only for males, while for females this lengthening is found only for the dyslectics, in contrast to a shortening of TP observed for the controls, and this is intriguing. Ingalhalikar et al. (2014) have recently shown that “In all supratentorial regions, males had greater within-hemispheric connectivity, as well as enhanced modularity and transitivity, whereas between-hemispheric connectivity and cross-module participation predominated in females. However, this effect was reversed in the cerebellar connections.” Can our results be related to these gender differences in cerebellar connectivity? There is much to explore here in future studies.

The current study is a preliminary attempt to examine the connection between sensorimotor training, TP and dyslexia. The main limitations of the current study are the small sample size and the use of only one training paradigm. In the future, a study on a larger sample that includes dyslexic no-training and verbal control groups may extend the current results.

## Conclusion/Significance

The current findings suggest that the combination of motor and mindful training, embedded in QMT, has a differential effect depending on one’s gender and whether one is dyslectic or not. This may have valuable implications for educational and contemplative neuroscience, in emphasizing the connection between specifically-structured motor training, time estimation and attention.

## Author Contributions

TDB-S and JG conceived and designed the experiments, analyzed the data and wrote the article.

## Conflict of Interest Statement

The authors declare that the research was conducted in the absence of any commercial or financial relationships that could be construed as a potential conflict of interest.
